# Mass spectrometric analysis of lipid A obtained from the lipopolysaccharide of *Pasteurella multocida*

**DOI:** 10.1039/d0ra05463a

**Published:** 2020-08-20

**Authors:** Abdul Tawab, Noor Akbar, Mujtaba Hasssan, Fazale Habib, Aamir Ali, Moazur Rahman, Abdul Jabbar, Waqar Rauf, Mazhar Iqbal

**Affiliations:** Health Biotechnology Division, National Institute for Biotechnology and Genetic Engineering (NIBGE) P.O. Box 577, Jhang Road Faisalabad-38000 Pakistan hamzamgondal@gmail.com +92 419201403; Department of Biotechnology NIBGE, Pakistan Institute of Engineering and Applied Sciences (PIEAS) Lehtrar Road Nilore Islamabad-45650 Pakistan; School of Biological Sciences, University of the Punjab Lahore Pakistan; Department of Biotechnology, Mirpur University of Science and Technology (MUST) Mirpur-10250 Azad Jammu and Kashmir Pakistan

## Abstract

Haemorrhagic septicaemia is mainly caused by an opportunistic pathogen, *Pasteurella multocida*, a major threat to the livestock dependent economies. The main endotoxins are lipopolysaccharides. The lipid A, a key pathogenic part of lipopolysaccharides, anchors it into the bacterial cell membrane. Hence, profiling of the lipid A is important to understand toxicity of this pathogen. Despite a significant progress made on glycan analyses of core regions of lipopolysaccharides from various *P. multocida* strains, the structure of lipid A has not been reported yet. The lipid A of *P. multocida* type B:2 was analyzed using ESI-MS/MS to identify the acylation patterns, number and length of various acyl fatty acids, phosphorylation level and lipid A modifications. The MS^*n*^ data revealed the existence of multiple lipid A variants, *i.e.* mono and bisphosphorylated hepta-, hexa-, penta- and tetra-acylated structures, decorated with varied levels of 4-amino-4-deoxy-l-arabinose (Ara4N) on C-1 and/or C-4′ phosphate groups of proximal and distal glucosamine lipid A backbone. The detailed mass spectrometric analyses revealed that even within the same class, lipid A exhibits several sub-variant structures. A primary and secondary myristoylation at C-2, C-3, C-2′ and C-3′ was observed in all variants except hepta-acylated lipid A that carried a secondary palmitate at C-2 position. The lipid A profiling described herein, may contribute in exploring the mechanisms involved in endotoxicity of *P. multocida* type B:2 in haemorrhagic septicaemia disease.

## Abstract 2

LC/MS-based variant profiling of lipid A component of endotoxic lipopolysaccharides of *Pasteurella multocida* type B:2, a causative agent of haemorrhagic septicaemia in water buffalo and cattle.

## Graphical abstract

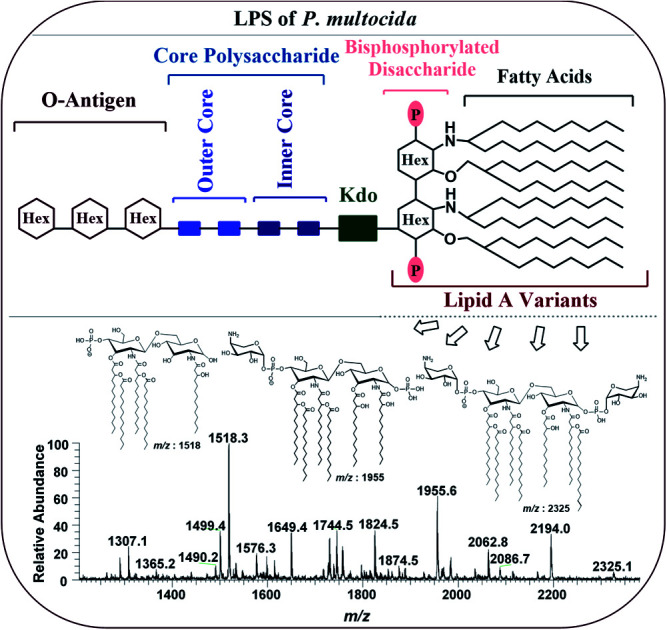

## Introduction

*Pasteurella multocida* is an opportunistic Gram-negative bacterial pathogen associated with various septic diseases of wild as well as domestic animals.^1^ Based on capsular chemistry, *P. multocida* strains have been classified into 5 sub-groups *i.e.* A, B, D, E and F; whereas, on the basis of the variations in their lipopolysaccharides (LPS), there are around 16 Heddleston serovars.^2,3^ Haemorrhagic septicaemia (HS) disease is mostly associated with serotypes B:2 and E:2 of *P. multocida* subsp. *multocida* (Carter and Heddleston system), corresponding to the 6:B and 6:E serotypes in Namioka–Carter system.^4^ HS is endemic in ungulates, predominantly water buffalo (*Bubalus bubalis*) and cattle in most parts of tropical Asia and Africa.^5,6^ In fact, HS is classified among list B cattle diseases notifiable to the Office International des Epizooties (OIE) with 100% mortality rate in infected animals if not treated at early stage.^7^ The diseased animals usually collapse and die within a few hours to a few days after the onset of the illness. Sudden death with few or no clinical signs can also be seen while the symptomatic animals, especially buffalo, rarely recover.^4,8^ This makes HS, the most important disease of bovines in terms of economic impact in various tropical and sub-tropical parts of the world.^9^ In Pakistan, having cattle and buffalo populations of 47.8 and 40 million respectively,^8^ young buffalo, being the most affected group,^10^ has been reported as an important reservoir of *P. multocida*.^4^

The pathogenesis of the *P. multocida* is poorly understood as compared to other Gram-negative bacteria. The haemagglutinin surface adhesin,^11^ capsular polysaccharides and LPS are critical virulence factors.^12^ The LPS plays a significant role in adhesion of *P. multocida* to the respiratory epithelium of host.^13^ The mutants attenuated to acapsular as well as truncated LPS, showed no or significantly reduced virulence and found to be more prone to the host antimicrobial peptides.^14–16^ Indeed, intravenous administration of the purified LPS induced the clinical signs resembling haemorrhagic septicaemia in buffalo *i.e.* endotoxic shock.^17^ Notably, in LPS, the lipid A part is considered as the active endotoxic part leading to inflammation, sepsis and septic shock in Gram-negative bacterial infections.^18,19^ Lipid A is primary pathogen-associated molecular pattern (PAMP) that binds to the pattern recognition receptors (PRR) *e.g.* toll-like-receptors (TLRs) of various eukaryotic cells. The first encounter of bacterial LPS occurs with a host cell plasma protein called lipopolysaccharide binding protein (LBP). LBP-bound LPS is recognized by a protruding CD14 receptor on the phagocyte cell membrane for onward presentation to TLR4. An intracellular signalling cascade, causing the induction of nuclear factor kappa-light-chain-enhancer of activated B cells (NF-κB), is triggered by this complex oligomerization. The downwards signalling leads to the NF-κB activation, pro-inflammatory cytokines production and induction of more and more host immune cells.^20–22^ Consequently, excessive inflammatory response of the innate immune system leads to sepsis and septic shock.^23^

The disaccharide backbone of lipid A is highly conserved among most of the bacterial species^24,25^ with few exceptions.^26,27^ The overall chemical structure of Gram-negative bacterial lipid A consists of disaccharide backbone of two covalently bonded pyranosidic hexaosamine residues (β-d-Glc*p*N4P-(1→6)-α-d-Glc*p*N1P). The skeletal disaccharide moiety is bonded to fatty acid chains at C-2/C-2′, C-3/C-3′, and mono or bisphosphorylation modifications at C-1, C-4′. The length of the fatty acid chains varies from C_10_ to C_16_ carbon atoms; however, some higher length chain may also exist.^28^ The endotoxicity of lipid A is associated with its structure *i.e.* number and length of fatty acyl chains, nature and phosphorylation of its glycan disaccharide backbone.^29–31^ The bisphosphorylated lipid A, substituted by six or seven asymmetrically distributed fatty acids linked *via* ester and amide linkages, is found to be highly endotoxic to the mammalian host.^32^ Such asymmetric (4/2) hexa-acylated and (4/3) hepta-acylated lipid A variants are reported in diverse pathogenic Gram-negative bacteria *e.g. Salmonella* Typhimurium,^28^*E. coli*,^33^*Klebsiella pneumoniae*,^34^*Hafnia alvei*.^35^ For intracellular survival, the Gram-negative bacteria modify their lipid A structures by adding phosphoethanolamine (PEtn),^36^ 4-amino-4-deoxy-l-arabinose (Ara4N), and palmitate residues.^37^ These modifications promote resistance against host antimicrobial peptides that are mobilized at the time of infection to destroy the invading microbes. Moreover, such changes help the pathogen to evade recognition by the innate immune receptor, TLR4.^38^

A significant progress has been made towards characterization of the polysaccharide components of LPS *i.e.* inner and outer core regions, produced by many *P. multocida* isolates belonging to serovars 1, 2, 3, 5, 9 and 14.^12,66–68^ Moreover, the outer glycan part of LPS obtained from *P. multocida* genome strain Pm70 has also been structurally characterised.^39^ However, despite its strong correlation with endotoxicity, the structure of lipid A part of the LPS of *P. multocida* has not yet been determined. Thus, in order to fill this knowledge gap, lipid A structure of pathogenic *P. multocida* type B:2 isolate (termed as PM2) from Pakistan was investigated using mass spectrometric technique that will help in better understanding of pathogenicity of this bacterium.

## Results

**Fig. 1:**
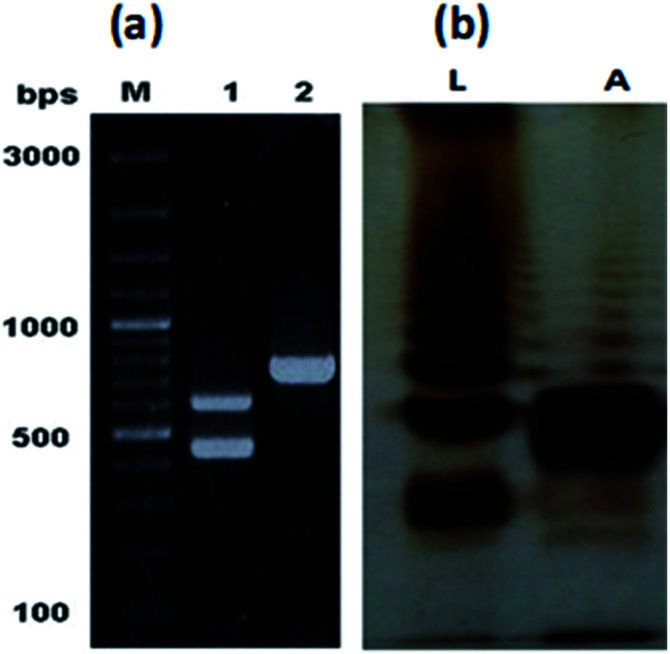
Molecular identification of PM2 and analysis of the purified LPS. (a) PCR based confirmation of PM2 isolate by 460 and 590 bp amplifications of *KMT1* and *6b* genes respectively (lane 1), and sub-serogroup B:2 type specific *bcbD* gene amplification of 758 bp (lane 2). (b) DOC-PAGE gel electrophoresis of the purified LPS of PM2. Lane L represents the control LPS of *S. Typhi* and lane A represents the precipitated fraction of the purified LPS of PM2.

The characteristic colonies of *P. multocida*: grayish, translucent and mucoid having around 1 mm diameter, were obtained on CSY agar plates. The molecular identification of the isolate resulted in amplification of 460 bp fragment of *KMT1* gene, 590 bp fragment of *6b* gene and 758 bp fragment of *bcbD* gene (Table 2), confirming the isolate identity as *P. multocida* belonging to B:2 type (Fig. 1a). No amplification was observed in case of negative controls. Large scale fermentation, resulted in 5 g L^−1^ yield of wet cell pellet and the LPS extraction yielded 55 mg of purified LPS per gram of bacterial cell pellet. The DOC-PAGE analysis showed the typical heterogenic patterns of the purified rough type LPS (Fig. 1b).

### Characterization of lipid A structure

#### ESI-MS/MS analysis of lipid A

**Fig. 2:**
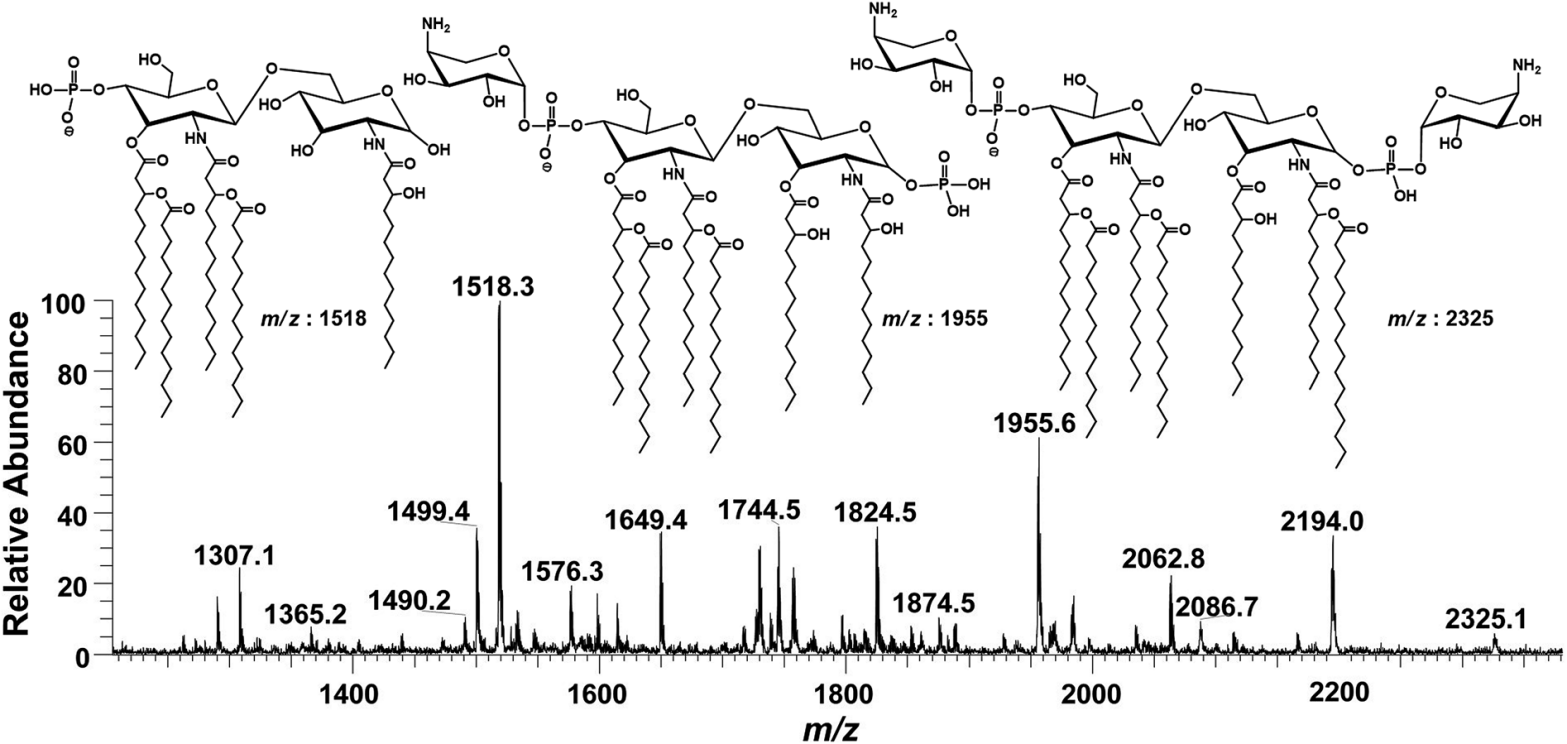
ESI-MS full scan of lipid A from PM2 in negative ionization mode exhibiting various structures.

The intact lipid A sample, obtained from the mild acid hydrolysis, was dissolved in the mixture of methanol and chloroform (1 : 1) and injected to ESI-MS using direct syringe pump. The full scan data at negative ion mode is shown in Fig. 2, which revealed that lipid A structure of *P. multocida* exhibited a high-level of heterogeneity, comprising of different fatty acyl chains and covalent modifications. Apparently, lipid A consists of hepta-, hexa-, penta- and a minor variant of tetra-acylation having 7, 6, 5 and 4 fatty acid acyl chains, respectively, attached to the glucosamine disaccharide backbone, which is mono and/or bisphosphorylated at C-1 and C-4′ positions with extended substitution(s) of one or two 4-amino-4-deoxy-l-arabinopyranose (Ara4N) molecules (Fig. 2).

Notably, these lipid A variants show highly capricious endotoxicity. Multiple lipid A structures complicate interpretation and can have variable effects on innate host immune response. The immunogenic properties of lipid A are directly correlated with the number of acyl chains, phosphate groups and covalent modifications, *i.e.*, the incorporation of palmitate, the addition of PEtN and/or Ara4N.^38^ Usually, bisphosphorylated asymmetric (4/2) hexa-acylated lipid A induces the strongest proinflammatory immune reactions after binding to TLR4, whereas, penta-acylated lipid A exhibits lower binding activity. Overall, bisphosphorylated versions of hepta-acylated (4/3), asymmetric hexa-acylated (4/2) and penta-acylated (3/2) lipid A activate NF-κB pathway and are considered as TLR4 expression agonists. Conversely, bisphosphorylated tetra-acylated (2/2) lipid A, monophosphorylated penta-acylated (3/2) and bisphosphorylated symmetric (3/3) hexa-acylated lipid A antagonize proinflammatory NF-κB pathway.^40,41^ Overall, endotoxicity of the pathogens *i.e. P. multocida*, is the collective impression of all these lipid A variants. Therefore, the determination of precise structure of lipid A species is important to visualize the endotoxicity as well as pathogenicity of any Gram-negative bacteria. Hence, the accurate structure of these lipid A variants of the tested *P. multocida* PM2 strain have been illustrated below in descending order, on the basis of their mass/charge (*m*/*z*) values and is summarized in Table 1. The conjugation sites were confirmed by correlating the acyl group fragmentations with the cross-ring fragmentations that are denoted in the manuscript according to the nomenclature proposed by Domon and Costello^42^ and Morrison *et al.*^43^

**Table 1:** Summary of mass spectral data of PM2 lipid A variants showing their ion abundance, phosphorylation levels, covalent modifications and fatty acyl chains

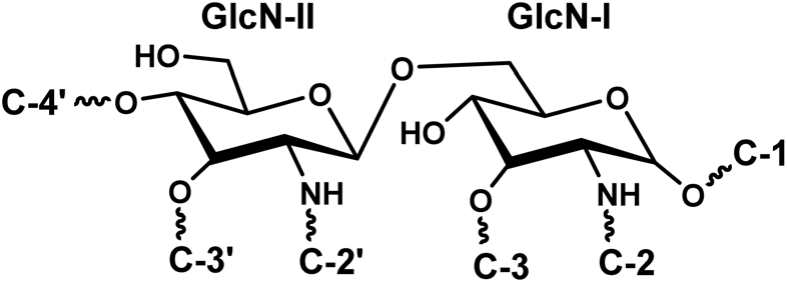								
S. #	*m*/*z*	RA^a^	C-4′	C-3′	C-2′	C-3	C-2	C-1
**Hepta-acylated**								
1	2325	05	Ara4N–P^b^	3-OH–C_14_ (C_14_)	3-OH–C_14_ (C_14_)	3-OH–C_14_	3-OH–C_14_ (C_16_)	P–Ara4N
2	2194	25	Ara4N–P	3-OH–C_14_ (C_14_)	3-OH–C_14_ (C_14_)	3-OH–C_14_	3-OH–C_14_ (C_16_)	P^c^
3	2166	06	Ara4N–P	3-OH–C_14_ (C_14_)	3-OH–C_14_ (C_12_)	3-OH–C_14_	3-OH–C_14_ (C_16_)	P
4	2114	08	Ara4N–P	3-OH–C_14_ (C_14_)	3-OH–C_14_ (C_12_)	3-OH–C_14_	3-OH–C_14_ (C_16_)	H
5	2063	22	P	3-OH–C_14_ (C_14_)	3-OH–C_14_ (C_14_)	3-OH–C_14_	3-OH–C_14_ (C_16_)	P
6	2035	07	P	3-OH–C_14_ (C_14_)	3-OH–C_14_ (C_12_)	3-OH–C_14_	3-OH–C_14_ (C_16_)	P
7	1983	13	P	3-OH–C_14_ (C_14_)	3-OH–C_14_ (C_12_)	3-OH–C_14_	3-OH–C_14_ (C_16_)	H

**Hexa-acylated**								
8	2087	15	Ara4N–P	3-OH–C_14_ (C_14_)	3-OH–C_14_ (C_14_)	3-OH–C_14_	3-OH–C_14_	P–Ara4N
9	1967	10	Ara4N–P	3-OH–C_14_ (C_14_)	3-OH–C_14_ (C_14_)	H	3-OH–C_14_ (C_16_)	P
10	1956	42	Ara4N–P	3-OH–C_14_ (C_14_)	3-OH–C_14_ (C_14_)	3-OH–C_14_	3-OH–C_14_	P
11	1927	05	Ara4N–P	3-OH–C_14_ (C_14_)	3-OH–C_14_ (C_12_)	3-OH–C_14_	3-OH–C_14_	P
12	1888	09	Ara4N–P	3-OH–C_14_ (C_14_)	3-OH–C_14_ (C_14_)	H	3-OH–C_14_ (C_16_)	H
13	1875	05	Ara4N–P	3-OH–C_14_ (C_14_)	3-OH–C_14_ (C_14_)	3-OH–C_14_	3-OH–C_14_	H
14	1853	05	P	3-OH–C_14_	3-OH–C_14_ (C_14_)	3-OH–C_14_	3-OH–C_14_ (C_16_)	P
15	1837	10	P	3-OH–C_14_ (C_14_)	3-OH–C_14_ (C_14_)	H	3-OH–C_14_ (C_16_)	P
16	1824	31	P	3-OH–C_14_ (C_14_)	3-OH–C_14_ (C_14_)	3-OH–C_14_	3-OH–C_14_	P
17	1797	15	P	3-OH–C_14_ (C_14_)	3-OH–C_14_ (C_12_)	3-OH–C_14_	3-OH–C_14_	P
18	1773	08	P	3-OH–C_14_	3-OH–C_14_ (C_14_)	3-OH–C_14_	3-OH–C_14_ (C_16_)	H
19	1756	21	P	3-OH–C_14_ (C_14_)	3-OH–C_14_ (C_14_)	H	3-OH–C_14_ (C_16_)	H
20	1744	20	P	3-OH–C_14_ (C_14_)	3-OH–C_14_ (C_14_)	3-OH–C_14_	3-OH–C_14_	H
21	1739	18	P	3-OH–C_14_ (C_14_)	3-OH–C_14_ (C_14_)	H	3-OH–C_14_ (C_16_)	–H_2_O
22	1726	20	P	3-OH–C_14_ (C_14_)	3-OH–C_14_ (C_14_)	3-OH–C_14_	3-OH–C_14_	–H_2_O
23	1717	09	P	3-OH–C_14_ (C_14_)	3-OH–C_14_ (C_12_)	3-OH–C_14_	3-OH–C_14_	H

**Penta-acylated**								
24	1888	09	Ara4N–P	3-OH–C_14_	3-OH–C_14_ (C_14_)	H	3-OH–C_14_ (C_16_)	P–Ara4N
25	1876	12	Ara4N–P	3-OH–C_14_	3-OH–C_14_ (C_14_)	3-OH C_14_	3-OH–C_14_	P–Ara4N
26	1860	04	Ara4N–P	3-OH–C_14_ (C_14_)	3-OH–C_14_ (C_14_)	H	3-OH–C_14_	P–Ara4N
27	1757	20	Ara4N–P	3-OH–C_14_	3-OH–C_14_ (C_14_)	H	3-OH–C_14_ (C_16_)	P
28	1745	27	Ara4N–P	3-OH–C_14_	3-OH–C_14_ (C_14_)	3-OH–C_14_	3-OH–C_14_	P
29	1729	20	Ara4N–P	3-OH–C_14_ (C_14_)	3-OH–C_14_ (C_14_)	H	3-OH–C_14_	P
30	1649	28	Ara4N–P	3-OH–C_14_ (C_14_)	3-OH–C_14_ (C_14_)	H	3-OH–C_14_	H
31	1614	12	P	3-OH–C_14_	3-OH–C_14_ (C_14_)	3-OH–C_14_	3-OH–C_14_	P
32	1598	11	P	3-OH–C_14_ (C_14_)	3-OH–C_14_ (C_14_)	H	3-OH–C_14_	P
33	1546	06	P	3-OH–C_14_	3-OH–C_14_ (C_14_)	H	3-OH–C_14_ (C_16_)	H
34	1534	10	P	3-OH–C_14_	3-OH–C_14_ (C_14_)	3-OH–C_14_	3-OH–C_14_	H
35	1518	100	P	3-OH–C_14_ (C_14_)	3-OH–C_14_ (C_14_)	H	3-OH–C_14_	H
36	1500	30	P	3-OH–C_14_ (C_14_)	3-OH–C_14_ (C_14_)	H	3-OH–C_14_	–H_2_O
37	1490	10	P	3-OH–C_14_ (C_14_)	3-OH–C_14_ (C_12_)	H	3-OH–C_14_	H

**Tetra-acylated**								
38	1439	05	Ara4N–P	3-OH–C_14_	3-OH–C_14_ (C_14_)	H	3-OH–C_14_	H
39	1308	18	P	3-OH–C_14_	3-OH–C_14_ (C_14_)	H	3-OH–C_14_	H
40	1290	14	P	3-OH–C_14_ (–H_2_O)	3-OH–C_14_ (C_14_)	H	3-OH–C_14_	H
41	1262	05	P	3-OH–C_14_ (–H_2_O)	3-OH–C_14_ (C_12_)	H	3-OH–C_14_	H

a RA = Relative abundance in full scan MS.

b Ara4N–P = 4-amino-4-deoxy-l-arabinose bonded with phosphate group.

c P = phosphate group.

#### Profiling of hepta-acylated lipid A variants

**Fig. 3:**
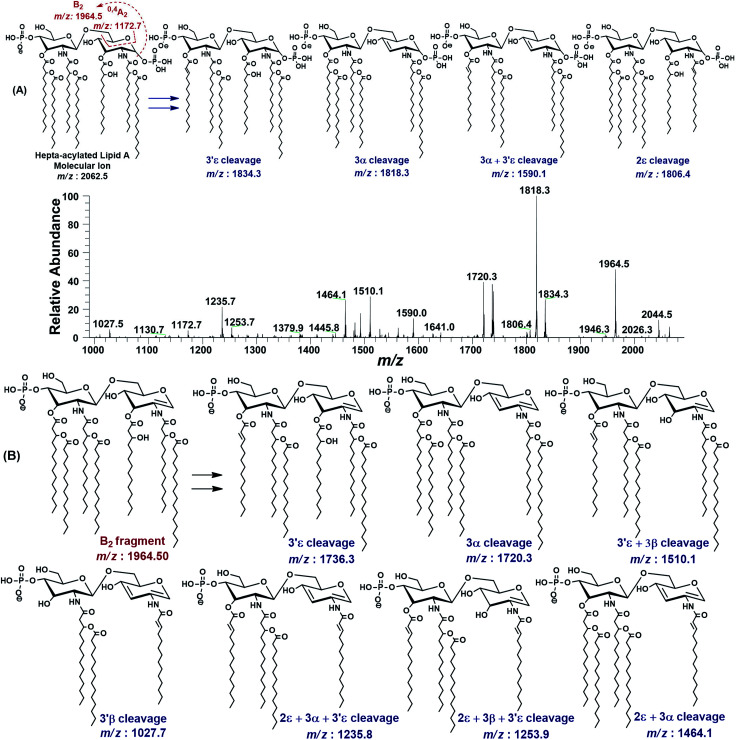
The MS^2^ of representative hepta-acylated lipid A molecular ion at *m*/*z* 2062.5 @CID 24, showing its fragmentation patterns. Red labels denote standard Domon and Costello fragments^42^ and blue labels denote the fragments arising from the loss of acyl chains that have been symbolized by their cleavage sites adopting the terminology proposed by Morrison *et al.*^43^ (A) Fragmentation (deacylation) while retaining H_3_PO_4_ at 1-position. (B) Simultaneous dephosphorylation and de-acylation.

Hepta-acylated lipid A structures exhibit seven variants in full scan spectrum at *m*/*z* 2325, 2194, 2166, 2114, 2063, 2035 and 1983. Their ion abundance, phosphorylation levels, covalent modifications and fatty acyl chains have been summarized in descending order in Table 1 (S. # 1–7). The structures of these variants have been determined through tandem mass spectrometric investigations. A detailed fragmentation analysis of one of the variants showing the ion peak at *m*/*z* 2062.5, is given here as a representative of this class (Fig. 3). The ESI-MS^2^ fragmentation (@CID 24) of the molecular ion at *m*/*z* 2062.5 yielded a series of daughter ions, resulting from the elimination of H_2_O, phosphate group and acyl chains from different positions. The daughter ion peaks at *m*/*z* 2044.5 and *m*/*z* 1964.5 were originated by the loss of a water and phosphoric acid (H_3_PO_4_) from the parent ion, respectively. Individual cleavages of myristate (C_14:0_) at 3′ε position, 3-hydroxymyristate [3-OH–C_14:0_] at 3α and palmitate (C_16:0_) at 2ε position yielded the fragments at *m*/*z* 1834.3, *m*/*z* 1818.3 and *m*/*z* 1806.4, respectively. The product ion at *m*/*z* 1834.3, generated after the 3′ε loss of a myristoyl group (*Δ* = 228 Da), from the secondary acyl chain of GlcN-II suggests a bisphosphorylated structure with fatty acyl chain ratio of 3/3 on both the GlcN-II : GlcN-I disaccharide backbone (Fig. 3A).

The base peak at *m*/*z* 1818.3 in MS^2^ spectrum of *m*/*z* 2062.5 was attributed to apparently the most stable daughter ion as a result of 3α elimination of 3-hydroxymyristate (244 Da) at GlcN-I leading to a bisphosphorylated fragment with fatty acyl chain distribution of 4/2 on GlcN-II : GlcN-I disaccharides, respectively. The fragment ion at *m*/*z* 1818.3 further lost the secondary acyl chain (C_14:0_) from the ester-linked 3-OH–C_14:0_ fatty acid at 3′ε position of GlcN-II, generating the subsequent fragment at *m*/*z* 1590.1, corresponding to a bisphosphorylated species with 3/2 fatty acyl chains on GlcN-II : GlcN-I disaccharides (Fig. 3A).

The minor peak at *m*/*z* 1806.5 was correlated with the concomitant 2ε loss of palmitate [C_16:0_] at the secondary acyl of GlcN-I. The low abundance of *m*/*z* 1806.5 is due to the fact that C_16:0_ is attached to the amide linked 3-OH–C_14:0_ at C-2 position. Secondary acyl fatty acids are strongly bonded with the amide linked 3-OH–C_14:0_ at C-2 and C-2′ positions on GlcN-I and GlcN-II, as compared to the ester linked primary acyl chains at C-3 and C-3′ positions.

The MS^2^ of *m*/*z* 2062.5 resulted in several additional peaks due to the formation of B_2_ fragment at *m*/*z* 1964.5 by the neutral loss of one phosphoric acid (H_3_PO_4_), followed by fatty acyl chains (Fig. 3B). The fragments at *m*/*z* 1736.3 and *m*/*z* 1720.3 were ascribed to 3′ε cleavage of myristic acid C_14:0_ and 3α cleavage of 3-OH–C_14:0_, respectively, from B_2_ fragment. The concurrent fragmentations of the B_2_ ion also exhibited the tendency for multiple cleavages of fatty acids. The fragments at *m*/*z* 1510.1 and *m*/*z* 1464.1 were produced by the loss of C_14:0_ at 3′ε site in conjunction with 3β cleavage of 3-OH–C_14:0_ and cleavage of 3-OH–C_14:0_ at 3α combined with that of C_16:0_ at 2ε position, respectively. Deacylation of three fatty acids yielded the ion peaks at *m*/*z* 1253.7 and *m*/*z* 1235.7. Similarly, removal of four fatty acids generated the ion peak at *m*/*z* 1027.7.

The MS^2^ fragmentation pattern of the precursor ion (*m*/*z* 2062.5) and its analysis suggested its structure as bisphosphorylated hepta-acylated disaccharide with 4/3 stoichiometric distribution of acyl chains. GlcN-I : GlcN-II disaccharide carries a 3-hydroxymyristate appendage at C-2 (primary position) attached *via* an amide linkage that further has an ester linkage with a palmitate (secondary acyl chain). The C-3 position also has a 3-hydroxymyristate moiety but through ester linkage. Another 3-hydroxymyristate is anchored at C-2′ (primary position) utilizing its hydroxyl group for ester linkage with a myristate (secondary). The C-3′ exhibits yet another 3-hydroxymyristate (primary position) through ester linkage with *O*-myristoylation (secondary) (Table 1, S. # 5). The cross-ring fragmentation represented as ^0,4^A_2_ at *m*/*z* 1172.7 further supported the distribution of different fatty acids (Fig. 3).

**Fig. 4:**
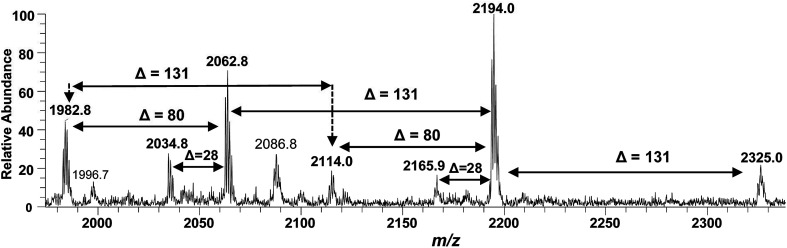
ESI-MS full scan of lipid A from PM2, in negative ionization mode, showing the correlation among hepta-acylated lipid A variants.

After confirming the structure of *m*/*z* 2062.5, the other hepta-acylated lipid A structures *i.e. m*/*z* 1982.8, 2034.8, 2114.0, 2165.9, 2194.0 and 2325.0 were successfully correlated as illustrated in Fig. 4. The ion peak at *m*/*z* 1982.8 was the monophosphorylated version of the structure proposed for *m*/*z* 2062.5 (*Δ* = 80 Da). The ion peak at *m*/*z* 2034.8 emerged due to loss of 28 Da (variation of ethylene group C_2_H_4_) from *m*/*z* 2063 indicating that one of the secondary myristoyl chains is replaced with laurate [C_12:0_]. Notably, almost all of the lipid A variants *i.e.* with hepta-, hexa-, penta- and tetra acylation, both in their monophosphorylated and bisphosphorylated forms and having covalent modification with Ara4N, demonstrated the substitution of myristate with laurate (reduction of *m*/*z* by 28 Da), suggesting a minor alternative lipid A variant. The exact position of laurate fatty acyl chain was determined to be 2′ through fragmentation of hexa-acylated precursor ion at *m*/*z* 1716.4 (Fig. 7).

The ion peak at *m*/*z* 2194 (Fig. 4) represented the addition of 131 Da to the bisphosphorylated hepta-acylated lipid A structure corresponding to the peak at *m*/*z* 2063, indicating that one of the phosphate group (more likely at 4′-position of GlcN-II) is decorated with Ara4N (*Δ* = 131 Da). The presence and position of mono and di-Ara4N on phosphate groups have been further confirmed in the following data analysis of hexa- and penta-acylated lipid A species and is summarized in Table 1. The ion peak at *m*/*z* 2194.0 exhibited the highest abundance among all of the hepta-acylated variants. The ion peaks at *m*/*z* 2165.9 and *m*/*z* 2114.0 demonstrated the reduction of 28 and 80 mass units from *m*/*z* 2194.0, indicating the subtraction of methylene and phosphate groups, respectively. The peak at *m*/*z* 2114.0 can be further interrelated with the peak at *m*/*z* 1982.8 by difference of 131 mass units (Ara4N).

Finally, the ion peak at *m*/*z* 2325 can be correlated by adding another 131 Da to *m*/*z* 2194.0 (2063 + 131 + 131 = 2325). The absence of any other peak between *m*/*z* 2194 and *m*/*z* 2325, especially the dephosphorylation possibility (2325 − 80 = 2245), indicated that both phosphate groups (1-position of GlcN-I and 4′-position of GlcN-II) of the entity corresponding to *m*/*z* 2325 are decked with Ara4N (Fig. 4 and Table 1, S. # 1). In short, the proposed structure for *m*/*z* 2325.0 comprises of a di-phosphorylated di-glucosamine backbone primarily acylated with 3-OH–C_14:0_ at the 2, 3, 2′, and 3′ positions and secondarily acylated with C_16:0_ at position 2, and with C_14:0_ at the 2′ as well as 3′ positions while both phosphate groups decorated with Ara4N.

#### Profiling of hexa-acylated lipid A variants

The current data revealed the presence of hexa-acylated lipid A variants of *P. multocida* with a high degree of heterogeneity in terms of the degree of acylation, phosphorylation and covalent modification with Ara4N, as has been summarized in Table 1. To develop the maximum correlation and illustration of the structures of different hexa-acylated lipid A variants, the fragmentation of three precursor ions at *m*/*z* 1955.3, *m*/*z* 1756.3 and *m*/*z* 1716.3 have been presented here. The remaining hexa-acylated ion peaks were successfully correlated with these fragmentation patterns.

**Fig. 5:**
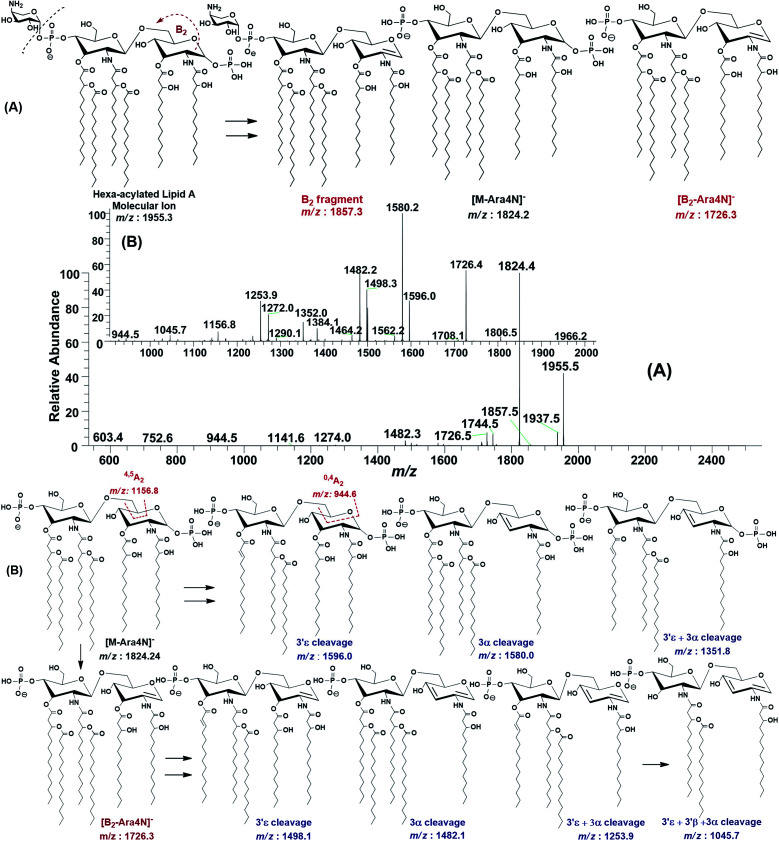
(A) ESI-MS^2^ of hexa-acylated lipid A molecular ion at *m*/*z* 1955.5 @CID 20, showing its fragmentation patterns (Ara4N ring is shown in small size), (B) MS^3^ of [M − Ara4N]^−^ fragment ion at *m*/*z* 1824.5 @CID 22, obtained from MS^2^ of *m*/*z* 1955.5.

The ESI-MS^2^ fragmentation of the ion at *m*/*z* 1955.3 (@CID 20) generated the daughter ions at *m*/*z* 1937.5, 1857.5, 1824.4, 1744.5, 1726.5 and 1482 along with a few minor peaks (Fig. 5A). The fragment at *m*/*z* 1937.5 [M − 18] conforms to the loss of H_2_O, while that at *m*/*z* 1857.3 to the B_2_ fragment formed after the removal of H_3_PO_4_ (*Δ* = 98 Da) from the C-1 position, causing unsaturation between C-1 and C-2 carbon atoms. Although the ion abundance of *m*/*z* 1857.3 is very low but its appearance gives an important hint about the presence of a stripped phosphate group at C-1 position without any covalent modification. The most abundant daughter ion at *m*/*z* 1824.4 corresponded to the elimination of Ara4N (*Δ* = 131 Da). This reflects that C-4′ phosphate group holds an Ara4N sugar as the covalent bond between the phosphate group and Ara4N sugar is among the most susceptible bonds. The removal of two moieties *i.e.* Ara4N and H_2_PO_3_ (*Δ* = 131 + 80) from *m*/*z* 1955.3, generated the ion peak at *m*/*z* 1744. Similarly, *m*/*z* 1726.4 was produced by the loss of Ara4N and H_3_PO_4_ (*Δ* = 131 + 98) (Fig. 5A). The removal of fatty acyl chains along with Ara4N were spotted in minor peaks. Due to highly labile nature of covalent bond between Ara4N and phosphate group (C-4′ position), the possible loss of the fatty acyl chains cannot be associated with the base peak at *m*/*z* 1824.4. This made us to pursue the MS^3^ of *m*/*z* 1824.4 to pinpoint the positions and number of fatty acyl chains.

After isolating *m*/*z* 1824.4 in ion trap, it was subjected to the MS^3^ fragmentation (@CID 22.0) that yielded multiple sub-fragments arising from the simultaneous loss of one or both of the phosphate groups (*m*/*z* 1726.4) and one or more of the fatty acyl chains (Fig. 5B). The ion peaks at *m*/*z* 1596.0 and *m*/*z* 1580.2 can be correlated to the loss of one C_14:0_ at 3′ε cleavage site and one 3-OH–C_14:0_ at 3α site, respectively, while losing both fatty acyl chains generated the peak at *m*/*z* 1352. The fragments at *m*/*z* 1498.1 and *m*/*z* 1482.1 were attributed to the loss of phosphate group along with the 3′ε cleavage of C_14:0_ and 3α cleavage of 3-OH–C_14:0_, respectively (Fig. 5B). The *m*/*z* 1272.0 and *m*/*z* 1253.9 were produced by the simultaneous loss of phosphate with one of the two pairs of fatty acids *i.e.* (i) C_14:0_ by 3′ε cleavage paired with 3-OH–C_14:0_ by 3α cleavage and (ii) C_14:0_ by 3′ε cleavage paired with C_14:0_ by 3β cleavage, respectively. The removal of phosphate together with cleavages at 3′ε, 3′β and 3α gave rise to the peak at *m*/*z* 1045.7. Similarly, the removal of four fatty acids produced the ion peak at *m*/*z* 1027.7.

The data analysis of MS^3^ fragmentation of the ion peak at *m*/*z* 1824.5 suggested its structure as bisphosphorylated hexa-acylated GlcN-I : GlcN-II disaccharide with 4/2 acyl chain stoichiometric distribution as 3-OH–C_14:0_ (C-2), 3-OH–C_14:0_ (C-3), 3-OH–C_14:0_ + C_14:0_ (C-2′) and 3-OH–C_14:0_ + C_14:0_ (C-3′) (Fig. 5B). The cross-ring fragments represented as ^0,4^A_2_ at *m*/*z* 944.5 and ^4,5^A_2_ at *m*/*z* 1156.8 further supported the distribution of different fatty acids. Out of the four primary chains, two have amide linkages at C-2 and at C-2′ positions, while the remaining two have ester linkages at C-3 and C-3′ positions of the core disaccharide skeleton as described in Table 1 (S. # 16).

Consequently, by combining the fragmentation data analysis of *m*/*z* 1955.3 (MS^2^) and *m*/*z* 1824.4 (MS^3^), the structure of the precursor at *m*/*z* 1955.3 was proposed as a bisphosphorylated, hexa-acylated lipid A variant of the *P. multocida* LPS. The C-1 and C-4′ positions are phosphorylated with an additional decoration of one covalently bonded Ara4N moiety at the phosphate group of C-4′ position. The acylation pattern looks to be 4/2 with four primary acyl chains. The two primary 3-OH–C_14:0_ chains are amide linked at C-2 and C-2′, while the rest are ester linked at C-3 and C-3′ positions of the GlcN disaccharide backbone moiety. The secondary acylation can be docked at the 3-hydroxy positions of primary fatty acid moieties attached at C-2′ and C-3′ locations (Table 1, S. # 10).

**Fig. 6:**
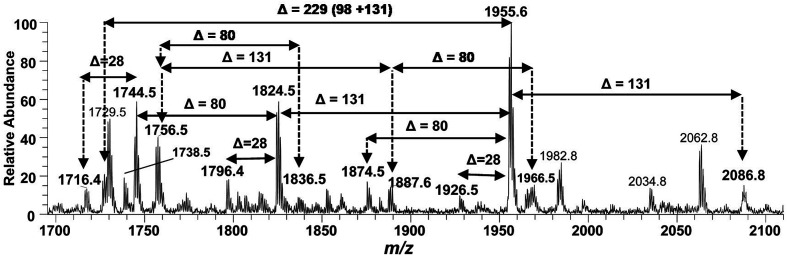
Negative ion mode ESI-MS full scan of lipid A from PM2, showing the correlation among hexa-acylated lipid A variants.

The structure elucidation of the precursor ion corresponding to *m*/*z* 1955.3 significantly contributed in assigning the other hexa-acylated ion peaks (Fig. 6). The parent ion at *m*/*z* 2086.8 represented the addition of a 131 Da unit, indicating that both of phosphate groups of bisphosphorylated hexa-acylated lipid A were decorated with Ara4N (Table 1, S # 8). The ion peak *m*/*z* 1926.5 emerged due to the loss of 28 Da (variation of ethylene group C_2_H_4_) from *m*/*z* 2063 suggesting that one of the C_14:0_ is replaced with C_12:0_ fatty acid possibly at C-2′ secondary position. Whereas, *m*/*z* 1874.5 was assigned to the mono dephosphorylated (*Δ* = 80 Da) version of *m*/*z* 1955.5. The precursor ions at *m*/*z* 1824.5 and *m*/*z* 1744.5 can be further interrelated with *m*/*z* 1955.5 by removing Ara4N (*Δ* = 131 Da) from C-4′ phosphate group and HPO_3_ (*Δ* = 80 Da) from C-1 positions, respectively (Fig. 6 and Table 1). Similarly, another hexa-acylated lipid A variant can be spotted at *m*/*z* 1726.5 by retreating Ara4N (*Δ* = 131) along with H_3_PO_4_ (*Δ* = 98).

**Fig. 7:**
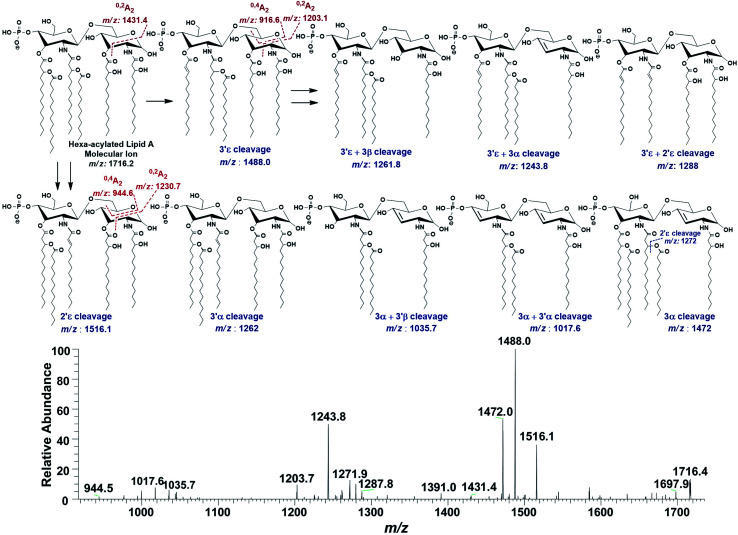
ESI-MS^2^ of hexa-acylated lipid A molecular ion at *m*/*z* 1716.4 @CID 20, showing its fragmentation patterns.

In the ion peak at *m*/*z* 1716.4, one of the secondary myristate was found to have been replaced with laurate (*Δ* = 28 Da). The exact position of C_12:0_ was determined by MS^2^ of *m*/*z* 1716.4, which yielded daughter ions by losing one of the fatty acyl chains at *m*/*z* 1516 (removal of laurate at 2′ε cleavage site), *m*/*z* 1488 (3′ε cleavage of myristate) and *m*/*z* 1472 (3α cleavage of 3-hydroxymyristate) (Fig. 7). The product ions arising from the elimination of two acyl chains were also detected at *m*/*z* 1288 [elimination of C_12:0_ (2′ε cleavage) together with C_14:0_ (3′ε cleavage)], *m*/*z* 1272 [elimination of C_12:0_ (2′ε cleavage) and β-OH–C_14:0_ (3α cleavage) as a ketene derivative], *m*/*z* 1261.8 [elimination of C_14:0_ (3′ε cleavage) and β-OH–C_14:0_ (3β cleavage)], and at *m*/*z* 1243.8 [elimination of C_14:0_ (3′ε cleavage) and 3-OH–C_14:0_ (3α cleavage)]. The plausible elimination of C_14:1_ both by charge-remote (loss of a free fatty acid) and charge-driven processes (loss of a ketene derivative) suggested its location at O-3′ as well as its secondary substitution by C_14:0_ (ion peak of *m*/*z* 1243.8).^44^

The product ions at *m*/*z* 1431.4, 1230.7 and *m*/*z* 1203.1 represented ^0,2^A_2_ fragments formed from the ions at *m*/*z* 1716.2, 1516.1 and 1488.0, respectively. Whereas, cross-ring fragments ^0,4^A_2_ at *m*/*z* 944.6 and *m*/*z* 916.6 were produced by *m*/*z* 1516 and 1488, respectively. These ions yielded by the in-source cross-ring fragmentation further established the distribution of the identified fatty acids on specific positions. After interpreting the types of fragment ions, it was possible to define fatty acid distribution on both GlcN residues. From this data analysis, it is suggested that the ion peak at *m*/*z* 1716.4 can be attributed to mono-phosphorylated, hexa-acylated lipid A form consisting of two GlcN, four primary acyl chains of β-OH–C_14:0_ at 2, 3, 2′ & 3′ positions and two secondary acyl chains C_12:0_ and C_14:0_ at 2′ and 3′ positions, respectively.

#### Profiling of hexa-acylated lipid A variant *m*/*z* 1756.3

**Fig. 8:**
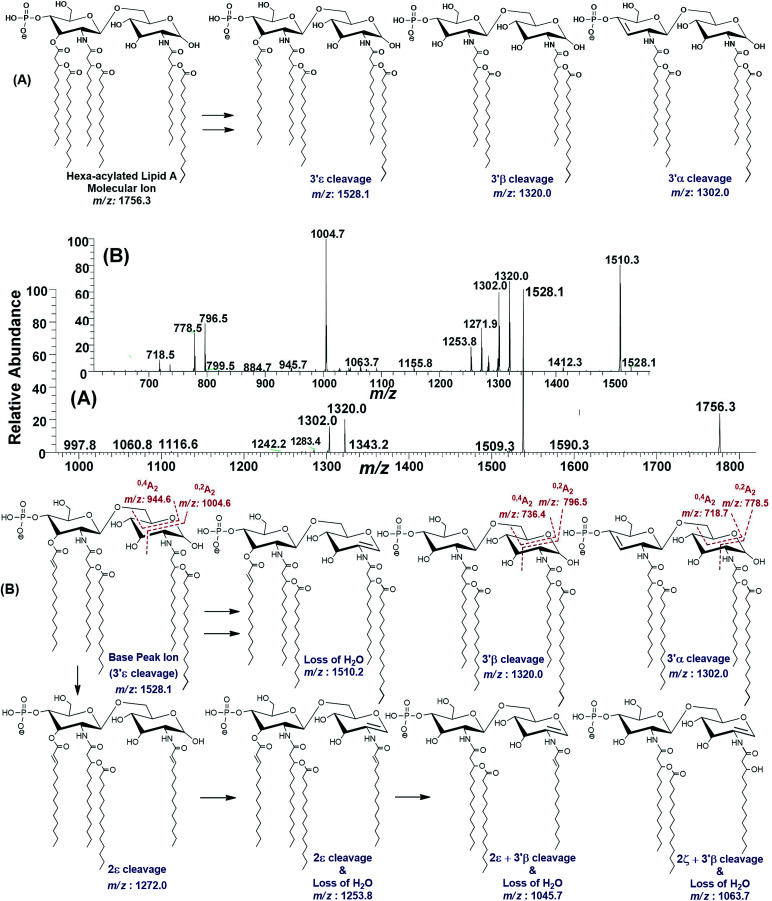
(A) ESI-MS^2^ of hexa-acylated lipid A molecular ion at *m*/*z* 1756.3 @CID 47, showing its fragmentation patterns, (B) MS^3^ of base peak at *m*/*z* 1528.5 @CID 22, obtained from MS^2^ of *m*/*z* 1756.3.

In the full scan MS data (Fig. 3), the ion at *m*/*z* 1756.3 (Table 1, S. # 19) with RA of 21% represents yet another hexa-acylated lipid A variant of *P. multocida* strain. The MS^2^ fragmentation of *m*/*z* 1756.3 generated daughter ions at *m*/*z* 1528.1, 1320 and 1302.0 (Fig. 8A). The loss of 228 mass units at from the parent *m*/*z* 1756.3, obtaining a stable base peak at *m*/*z* 1528.1, indicated the removal of a secondary acyl group C_14:0_ from the β-hydroxy acyl chain at 3′ε position rendering the remaining structure unsaturated. The cleavage of both primary and secondary acyl groups from the C-3′ of GlcN-II at 3′β position through charge-driven process (leaving OH behind) produced a fragment at *m*/*z* 1320.0, while further fragmentation at 3′α cleavage site through charge-remote process generates a product ion of *m*/*z* 1302 with unsaturation between C-3′ and C-4′ of GlcN-II^44^ (Fig. 8A).

The MS^3^ of the base peak *m*/*z* 1528.1, obtained from *m*/*z* 1756.3, generated product ions at *m*/*z* 1510, 1320, 1302, 1272, 1254, 1064, 1046, 1005, 946, 796, 778 and 718 (Fig. 8B). The ion at *m*/*z* 1510 was generated due to the water loss presumably C-1 on GlcN-I, resulting in an unsaturation between C-1 and C-2 atoms. The notion can be justified by the presence of *m*/*z* 1004.6, and absence of *m*/*z* 986.6 in case of water loss from the C-3 position of GlcN-I, due to cross-ring ^0,2^A_2_ fragmentation. The presence of both *m*/*z* 1320.0 and 1302.0 are indicative of the charge-driven as well as charge-remote type 3′α and 3′β cleavages entailing C-3′/C-4′ positions. The explanation is also supported by the presence of other ions at *m*/*z* 796.5, 778.5 (^0,2^A_2_) as well as at 736.4 and 718.7 (^0,4^A_2_) as a result of cross-ring fragmentations. The presence of *m*/*z* 1272.0 indicated the loss of a unit with *Δ* = 256 Da reflecting the elimination of C_16:0_ from 2ε position. Similarly, the generation of a peak at *m*/*z* 1253.8 can be spotted due to the loss of water from C-1 position along with the secondary deacylation from C-2 (2ε cleavage) (Fig. 8B). A less frequent 2ζ cleavage was also observed in combination with 3′β cleavage and water loss from C-1 leading to a product ion at *m*/*z* 1063.7.

The ESI-MS^2^ spectrum of *m*/*z* 1756.3 followed by MS^3^ of its daughter ion at *m*/*z* 1528.1 revealed its structure as hexa-acylated mono-phosphorylated variant with three primary acylations of 3-OH–C_14:0_ at C-2, C-2′ and C-3′ positions. The first two of these primary acyl chains are amide linked while the remaining one is ester linked. The secondary acylation of C_14:0_ was also observed at C-2′ and C-3′ while a C_16:0_ at C-2 position. The successful profiling of *m*/*z* 1756.3 contributed in correlating the peaks at *m*/*z* 1836 and 1887.6 in full scan data (Fig. 6): the addition of 80 and 131 mass units suggested the substitution of HPO_3_ at C-1 and Ara4N decoration on phosphate at C-4′ positions, respectively.

#### Profiling of penta- and tetra-acylated lipid A variants

**Fig. 9:**
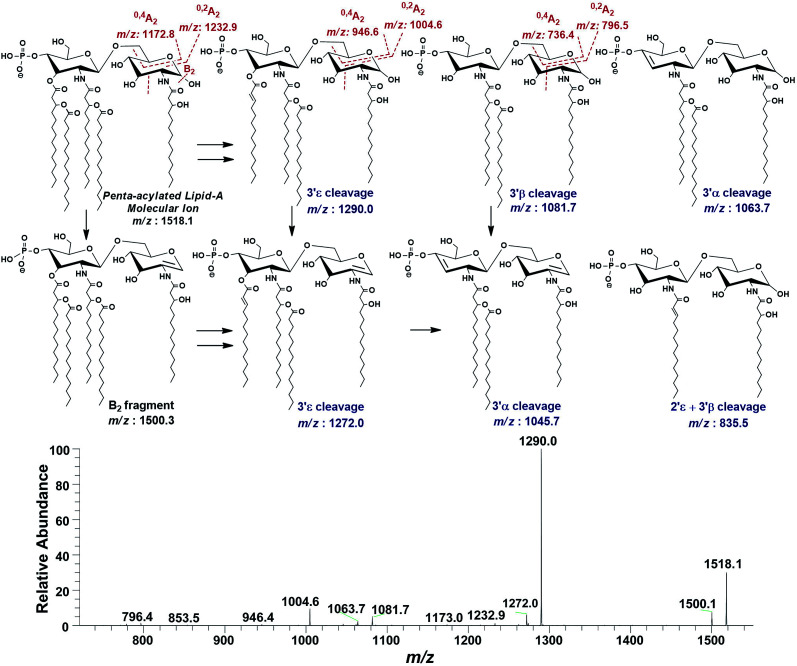
ESI-MS^2^ of penta-acylated lipid A molecular ion at *m*/*z* 1518.1 @CID 20, showing its fragmentation patterns.

The full scan MS data also revealed the presence of penta-acylated lipid A variants in LPS of *P. multocida*. Out of these, the prominent precursor ion at *m*/*z* 1518.1 was subjected to MS^2^ fragmentation, which produced the daughter ions at *m*/*z* 1500.3 (*Δ* = 18 Da), 1290.0 (*Δ* = 228 Da), 1272.0 (*Δ* = 228 + 18 = 246 Da) as a result of the loss of water from C-1 position (B_2_ ion) and cleavage of C_14:0_ at 3′ε, and both (Fig. 9). The fragment ions at *m*/*z* 1081.7 and 1063.7 appeared after the 3′β and 3′α cleavages of both primary and secondary acyl chains through charge-driven and charge-remote processes.^44^ The fragments at *m*/*z* 1272.0 and 1045.7 are the dehydrated versions of the fragments with *m*/*z* 1290.0 and 1081.7, respectively, as depicted in Fig. 9. The fragmentation data revealed the structure corresponding to the precursor at *m*/*z* 1518.1 as a mono-phosphorylated penta-acylated lipid A having 3-hydroxymyristate as primary acyl chains at C-2, C-2′ and C-3′ and C_14:0_ as secondary acyl chains at C-2′ and C-3′ positions (Fig. 9 and Table 1, S. # 35). The distribution of fatty acids was corroborated by cross-ring fragments ^0,2^A_2_ (*m*/*z* 1232.9, 1004.6 and 796.5) and ^0,4^A_2_ (*m*/*z* 1172.8, 946.6 and 736.4).^24^

**Fig. 10:**
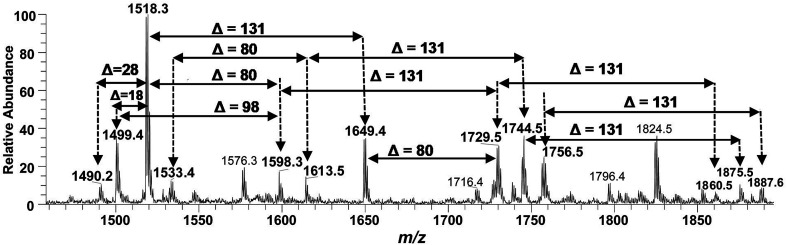
Negative ion mode ESI-MS full scan of lipid A from PM2, showing the correlation among penta-acylated lipid A variants.

Successful profiling of *m*/*z* 1518.1 contributed in correlation of other penta-acylated lipid A variants (Fig. 10). The ion at *m*/*z* 1490.2 (*Δ* = 28 Da) indicated a variation in fatty acid ruler such that a laurate (C_12:0_) is present at secondary acyl position of C-2′ with 4/1 fatty acyl distribution as described in the analysis of *m*/*z* 1518.1 (Table 1, S. # 36). There could also be a dehydrated moiety at *m*/*z* 1499.4 owing to the water loss from the anomeric C-1 position of the ion corresponding to the base peak at *m*/*z* 1518.0 (Table 1, S. # 35). The addition of HPO_3_ and Ara4N (*Δ* = 80 and 131 Da) to the base peak at *m*/*z* 1518.1 gave rise to the ions at *m*/*z* 1649.4 and *m*/*z* 1598.3, respectively (Table 1, S. # 30 & 32), which further proceeded to *m*/*z* 1729.5 after the addition of another set of HPO_3_ (1649 + 80 = 1729 Da) and Ara4N (1598 + 131 = 1729 Da) (Table 1, S. # 29).

The ion peak at *m*/*z* 1533.4 corresponded to another set of mono-phosphorylated penta-acylated lipid A molecules having 3-OH–C_14:0_ appendages at C-2, C-3, C-2′ and C-3′ positions while only one secondary C_14:0_ acyl chain at C-2′. Its bisphosphorylated (*Δ* = 80 Da) and Ara4N (*Δ* = 80 + 131 Da) analogues were spotted at *m*/*z* 1614 and 1745, respectively (Table 1, S. # 31 & 28). Similarly, there was another ion peak at *m*/*z* 1757 (Table 1, S. # 27), which indicated penta-acylation with 3/2 pattern and an Ara4N moiety on C-4′ phosphate group and a palmitate at secondary position of C-2. By the addition of 131 Da, a second Ara4N on C-1 phosphate group was also observed at *m*/*z* 1887.6 (Table 1, S. # 24). Another 3/2 penta-acylated lipid A appeared at *m*/*z* 1546 having only one phosphate group at C-4′. The ESI-MS/MS data also depicted a small proportion of tetra-acylated lipid A variants at *m*/*z* 1439, 1308, 1290 and 1262 (Table 1, S. # 38–41).

## Discussion

Haemorrhagic septicaemia (HS) is associated with *P. multocida* (serotype B:2 and E:2) infections in cloven-hoofed ungulates.^45^ The disease occurs in acute, sub-acute and chronic forms. Its initial phase includes temperature elevation, a stage of respiratory involvement and the terminal phase involves septicaemia and recumbence leading to death.^9^ Although the HS disease etiology is strongly related to the endotoxicity of lipid A component of the bacterial LPS, the structure elucidation of the lipid A has not been reported yet.

ESI-MS analysis of lipid A obtained from *P. multocida* isolate PM2 in current study, showed a high degree of complexity and heterogeneity. The overall heterogeneity of lipid A varies from hepta-acylated to tetra-acylated variants decorated with varied level of Ara4N moiety on C-1 and/or C-4′ phosphate groups of proximal and distal glucosamine lipid A backbone. The full scan mass spectrometric data revealed that even within the same class of lipid A, several sub-variant structures exist, which have been summarized in Table 1.

The hepta-acylated lipid A consists of seven structures with 4/3 acyl distribution on GlcN-II : GlcN-I sugar moieties of lipid A backbone, respectively (Table 1, S. # 1–7). All of these structures have four β-hydroxy C_14:0_ primary acyl chain, palmitate secondary acyl chain at C-2 position and myristate at C-3′ position. The secondary acyl chain at C-2′ position in majority of the hepta-acylated variants is C_14:0_, whereas, C_12:0_ is also found as a minor alternative (Table 1, S. # 3 and 6). Majority of hepta-acylated lipid A structures are bisphosphorylated (Table 1, S. # 1–3, 5 & 6). Ara4N was mostly found to decorate only C-4′ phosphate group (Table 1, S. # 2–4) except that the entity at *m*/*z* 2325 exhibits two Ara4N moieties at both phosphate groups. Similar to other Gram-negative bacteria, the palmitoylation as well as regulated addition of Ara4N in PM2 isolate may be environment-dependent and can contribute in protecting the bacterium from certain host immune defenses.^38^

Addition of palmitate fatty acyl chain to lipid A is catalyzed by PagP, a palmitoyl transferase enzyme, which is the component of PhoP–PhoQ regulatory system.^46^ The *pagP* gene and its functional homologs are distributed among a narrow group of primary pathogenic bacteria (*Salmonella* species,^47,48^*E. coli*,^33^*Proteus mirabilis*,^49^*Acinetobacter baumannii*,^50^*Pseudomonas aeruginosa*,^51^*L. pneumophila*, *B. bronchiseptica*, *Y. enterocolitica*, and *Y. pseudotuberculosis*^52^) and its regulation is often correlated with their pathogenic lifestyle.^53^ The addition of palmitate not only strengthens the outer membrane of the bacterial cell wall through the hydrophobic and van der Waals interactions, which prevents translocation of the cationic antimicrobial peptides across the bilayer, but also contributes in evading the host immune response by lowering TLR4 related signal transduction pathway as compared to hexa-acylated lipid A variants.^53^ Similarly, insertion of Ara4N is catalyzed by ArnT through activation of PmrA transcription factor. Being positively charged at neutral pH, Ara4N can neutralize the phosphate groups, consequently, reducing bacterial susceptibility to the cationic host antimicrobial peptides and polymyxin antibiotics.^38^

The hexa-acylated lipid A of the PM2 isolate exhibited a high degree of variation with most of the structures bisphosphorylated at C-1 and C-4′ positions. In five structures, phosphates were decorated with Ara4N at C-4′ positions and only one structure demonstrated Ara4N sugars on both of its phosphate groups (Table 1: S. # 8–23). There was a significant heterogeneity of fatty acyl patterns with respect to their relative positions and variation in chain length of fatty acids. Most of the hexa-acylated structures exhibited 4/2 fatty acyl stoichiometry with C_14_ carbon ruler at primary and secondary acyl chains. The presence of laurate (C_12:0_) at secondary acyl chain at C-2′ position was also spotted in minor proportion (Table 1: S. # 11, 17 and 23).

The bisphosphorylated 4/2 structures have been reported to warrant maximal immunostimulatory activities of LPS.^54,55^ In *P. multocida* infections these structures presumably induce high inflammatory response of the innate immune system, thus playing a pivotal role in causing HS. However, hexa-acylated structures having 4/2 fatty acyls also exist in which palmitate occupy secondary position at C-2 with absence of 3-OH–C_14:0_ at C-3 positions (Table 1: S. # 9, 12, 15, 19 and 21). The outer membrane-bound lipid A 3-*O*-deacylase, encoded by the *pag*L gene removed the fatty acyl chain from C-3 position of hepta-acylated lipid A. The PagL enzyme was initially reported in *Salmonella*, but later its homologs were found to be widely distributed among pathogenic and non-pathogenic Gram-negative bacterial species.^33^ The presence of hexa-acylated lipid A variants without fatty acyl chains at C-2 position hints the presence of PagL in *P. multocida* as well. The addition of palmitate at C-2 by PagP and the removal of primary acyl chain at C-3 by PagL have been reported to get activated by cationic antimicrobial peptides induced through the PhoP transcription factor.^46^ Hence, these structures are likely to contribute in evading the host immune defense in *P. multocida* infections as well. Moreover, hexa-acylated lipid A structures with 3/3 stoichiometry (Table 1: S. # 14 and 18), were also spotted in a minor proportion. Rather than acting as agonist, the 3/3 stoichiometric structures have been found to antagonize TLR4 receptor.^40^

Like hexa-acylated lipid A, penta-acylated class also exhibited several variants and most of those were bisphosphorylated with Ara4N placement only at C-4′ position in four structures, while three structures displayed Ara4N sugars on both of its phosphate groups (Table 1: S. # 24–37). Significant heterogeneity was found in the positions and types of fatty acyl chains with predominant 4/1 stoichiometry (Table 1: S. # 26, 29, 30, 32, 35–37) as demonstrated by a base peak at *m*/*z* 1518. Fatty acid distribution with 3/2 stoichiometry demonstrated two sub-categories, one with secondary palmitate at C-2 (Table 1: S. # 24, 27 and 33), while the second contained two primary acyl chains at C-2 and C-3 positions (Table 1: S. # 25, 28, 31 and 34). In small proportion, mono-phosphorylated tetra-acylated variants having 3/1 stoichiometry were also spotted (Table 1: S. # 38–41) in the lipid A of the PM2 isolate. TLR4 activation has been markedly decreased by tetra- and penta-acylated lipid A structures as compared to hexa-acylated variants. Even antagonistic trend to the TLR4 activity has been found in penta-acylated lipid A having 3/2 fatty acyl distribution stoichiometry lacking phosphate at C-1 position, as well as in bisphosphorylated tetra-acylated lipid A structures.^40,56^ Gram-negative bacteria modulate lipid A structures to evade host immune response *e.g. H. pylori* synthesizes a hexa-acylated lipid A intrinsically, but trims it down to tetra-acylated by the action of deacylase enzymes and also removes phosphates (by *Lpx*E & *Lpx*F) to switch the biological activity from TLR4 agonistic to antagonistic, which can turn its infection to a chronic one for the duration of host life.^57^

The lipid A of PM2 depicts a variety of acylation, and other modes of modifications *e.g.* phosphorylation and Ara4N decoration. The presence of both phosphate and Ara4N moieties can be located on C-1 and/or C-4′ positions of the basic glucosamine backbone. The ESI-MS^*n*^ further reveals the C-2, C-3, C-2′ and C-3′ bonded with primary and secondary myristoylation except few lipid A variants, where there is palmitoylation at C-2 position (Table 1, S # 1–7, 9, 12, 14, 15, 18, 19, 21, 24, 27 and 33) and lauric acid at secondary positions of C-2′ carbon (Table 1, S # 3, 4, 6, 7, 11, 17, 23, 37 and 41). There could be a strong correlation between the heterogeneous lipid A modification capabilities of *P. multocida* and its sporadic HS epidemics, which needs further investigations.

In addition to the number and length of the acyl groups, the stereochemistry and degree of their unsaturation may also have important biological implications. In our study, all the acyl groups bonded to the disaccharide backbone of lipid A happened to be saturated (Table 1), but in many other microbial strains, the unsaturated fatty acids may also exist.^58^ The carbon–carbon double bond on these unsaturated acyl groups can be located *via* a recently developed technique using visible-light activated [2 + 2] cycloaddition reaction and tandem mass spectrometry.^59^

## Experimental

### Material and method

#### Bacterial identification

*P. multocida* isolate, named PM2 was taken from Veterinary Research Institute, Peshawar and was revived in mice at Animal House Facility, National Institute for Biotechnology and Genetic Engineering (NIBGE), Faisalabad, Pakistan after approval from Institutional Bioethics Committee, ref. no: NIBGE/Bioethics/2014/02. The mice blood was directly streaked on casein sucrose yeast (CSY) agar plate and kept overnight at 37 °C. A well isolated colony having typical morphology of *P. multocida* was inoculated into 3 mL of CSY broth and incubated at 37 °C for overnight and preceded for molecular confirmation by polymerase chain reaction (PCR). Briefly, the DNA of overnight bacterial culture was extracted using genomic DNA purification kit (Thermo Scientific # K0512). Agarose gel electrophoresis (1%) and Nano-Drop (Thermo Scientific Nano-Drop 20000c) were used to check the integrity and quantification of the DNA respectively. The PCR of the extracted DNA was performed targeting two genes: *KMT1* gene fragment of 460 base pairs (bp), specific for *P. multocida* and a 590 bp fragment of *6b* gene, specific for its serotype. To identify its sub serogroup B:2 type, the 758 bp *bcbD* gene was also targeted.^60,69^ The composition of PCR mixture and thermal cycler conditions were kept same as reported earlier.^61^ The sequences of the primers used are mentioned in Table 2.

**Table 2:** Primer sequences used in the study

Primers	Oligonucleotide sequence (5′-3′)	Targeted gene	Amplicon size (bp)
PMPCR-F	ATCCGCTATTTACCCAGTGG	*KMT1*	460
PMPCR-R	GCTGTAAACGAACTCGCCAC		
KTT72	AGGCTCGTTTGGATTATGAAG	*6b*	590
KTSP61	ATCCGCTAACACACTCTC		
CAPB-F	CATTTATCCAAGCTCCACC	*bcbD*	758
CAPB-R	GCCCGAGAGTTTCAATCC		

### LPS extraction and purification

The PCR confirmed PM2 isolate was grown in sterile CSY broth using 6 × 2 L flasks at 37 °C and 180 rpm. After overnight growth, the cells were treated with formalin (0.5% v/v) for 2 hours and then harvested by 7000 rpm centrifugation at 10 °C. The wet cell biomass was subjected to LPS extraction by hot-phenol method.^62^ The extracted LPS were dialyzed for 3 consecutive days against deionized water using ZelluTrans/Roth dialysis membrane (T2 MWCO 6000–8000 cat # E665.1), with 2 water changes daily and lyophilized. The LPS were analyzed by deoxycholate-polyacrylamide gel electrophoresis (DOC-PAGE) followed by silver staining.^63,64^

### Acid hydrolysis and extraction of lipid A

For lipid A extraction, the purified LPS was subjected to mild acid hydrolysis.^65^ Briefly, 10 mg L^−1^ of the purified LPS was dissolved in 1% glacial acetic acid and heated at 95 °C using water bath for 90 minutes. The solution was cooled and ultracentrifuged at 96 000*g* at 4 °C for 5 hours with a drop of 1 M CaCl_2_. The pellet was dissolved in chloroform : methanol : water solution as 2 : 1 : 1 (v/v/v) ratio and kept overnight. The chloroform extract was dried under nitrogen and redissolved in chloroform : methanol : acetonitrile solution as 3 : 1 : 1 (v/v/v) ratio and used for Electron Spray Ionization (ESI) mass spectrometric analysis.

### Mass spectrometry of lipid A

The ESI-MS/MS was conducted using LTQ XL mass spectrometer (Thermo Electron Corporation, USA). The sample was analyzed through direct injection to the ESI probe set at negative ionization mode by syringe pump at a flow rate of 0.3 mL min^−1^. The temperature of capillary was kept at 198 °C. After getting the full scan mass spectrum data in the range of *m*/*z* 100 to *m*/*z* 3000, the generated ions were isolated in the ion trap and fragmented by collision induced dissociation (CID) energies ranging from 10 to 40 according to the stability of the target precursor ions selected for tandem mass spectrometry.

### Data analysis

The data was analysed using Xcalibur 2.2 and Chemdraw softwares. The identification was conducted using MS/MS fragmentation patterns of various precursor ions. The details for each specific fragmentation can be seen in the footnotes of all the corresponding figures.

## Conclusion

The pathogenic *P. multocida* PM2 isolate was found to exhibit a variety of lipid A variants, *i.e.* mono and bisphosphorylated hepta-, hexa-, penta- and tetra-acylated versions with the 4/3, 4/2, 3/3, 3/2, 4/1 and 3/1 patterns on the Glc-II and Glc-I backbone, respectively. It can also decorate its lipid A with the Ara4N sugar moiety, either at one or both of the phosphate moieties at C-1 and C-4′ positions. Majority of the lipid A variants are hexa-acylated with a total of 16 observed variants, followed by 14 penta, 7 hepta and 4 tetra-acylated variants. Based on the relative abundance, the penta-acylated version at *m*/*z* 1518.1 is the most abundant, followed by hexa-acylated lipid A variant having an *m*/*z* 1955.5. The presence of highly endotoxic hexa-acylated lipid A variants may have a leading role in inducing the disease, the haemorrhagic septicaemia. While covalently modified hexa-acylated lipid A variants (palmitoylation at C-2 position and Ara4N placement at C-4′ and/or C-1 phosphate positions) can contribute in evading the host immune responses.

## List of abbreviations

LPS: Lipopolysaccharides
ESI-MS: Electron spray ionization mass spectrometry
Ara4N: 4-Amino-4-deoxy-l-arabinose
HS: Haemorrhagic septicaemia
OIE: Office International des Epizooties
PAMP: Pathogen-associated molecular pattern
PRR: Pattern recognition receptors
TLRs: Toll-like-receptors
LBP: Lipopolysaccharide binding protein
NFκB: Nuclear factor-κB
PEtn: Phosphoethanolamine
CSY: Casein sucrose yeast
PCR: Polymerase chain reaction
DOC-PAGE: Deoxycholate-polyacrylamide gel electrophoresis
CID: Collision induced dissociation
RA: Relative abundance

## Conflicts of interest

The authors declare no conflict of interests and no permission is required for publication.

## Supplementary Material

## References

[cit1] Wilkie I. W., Harper M., Boyce J. D., Adler B. (2012). Pasteurella multocida: siseases and pathogenesis. Microbiol. Immunol..

[cit2] Carter G. R. (1952). The type specific capsular antigen of Pasteurella multocida. Can. J. Med. Sci..

[cit3] Heddleston K. L., Gallagher J. E., Rebers P. A. (1972). Fowl cholera: gel diffusion precipitin test for serotyping Pasteruella multocida from avian species. Avian Dis..

[cit4] Spickler A. R. , Hemorrhagic septicemia, in Factsheet, 2019, pp. 1–6, available from http://www.cfsph.iastate.edu/Factsheets/pdfs/hemorrhagic_septicemia.pdf

[cit5] Dagleish M. P., Hodgson J. C., Ataei S., Finucane A., Finlayson J., Sales J. (2007). *et al.*, Safety and protective efficacy of intramuscular vaccination with a live aroA derivative of Pasteurella multocida B:2 against experimental hemorrhagic septicemia in calves.. Infect. Immun..

[cit6] Wilson M. A., Rimler R. B., Hoffman L. J. (1992). Comparison of DNA fingerprints and somatic serotypes of serogroup B and E Pasteurella multocida isolates. J Clin Microbiol.

[cit7] Srivastava S. K. , Kumar A. A., Chaudhuri P. and Yadav M. P., Haemorrhagic septicaemia; manual of diagnostic tests and vaccines for terrestrial animals: mammals, birds and bees, in Terrestrial Manual, Office International Des Epizooties (OIE), Paris, France, 2008, vol. 2, pp. 739–751, http://www.oie.int/fileadmin/Home/eng/Health_standards/tahm/2.04.12_HS.pdf

[cit8] Wasti S. E. , Hanif S., Asif M., Malik M. S., Husnain Z. and Khatoon S., *et al.*, Pakistan Economic Survey 2018-19, in Pakistan Economic Survey 2018-19, Economic Adviser's Wing, Finance Division, Government of Pakistan, Islamabad, 2019, p. 27, available from http://finance.gov.pk/survey/chapters_19/Economic_Survey_2018_19.pdf

[cit9] Shivachandra S. B., Viswas K. N., Kumar A. A. (2011). A review of hemorrhagic septicemia in cattle and buffalo. Anim. Health Res. Rev..

[cit10] Ahmad M., Aziz M., Tunio M. T., Rehman R. u., Rehman A., Rehman K. u. (2018). *et al.*, Seroprevalence of hemorrhagic septicemia in buffalo and cattle in flood, irrigated and sandy areas of Punjab, Pakistan. Pure Appl. Biol..

[cit11] Fuller T. E., Kennedy M. J., Lowery D. E. (2000). Identification of Pasteurella multocida virulence genes in a septicemic mouse model using signature-tagged mutagenesis. Microb. Pathog..

[cit12] Harper M., Boyce J. D. (2017). The myriad properties of Pasteurella multocida lipopolysaccharide. Toxins.

[cit13] Gallego C., Romero S., Esquinas P., Patiño P., Martínez N., Iregui C. (2017). Assessment of Pasteurella multocida a lipopolysaccharide, as an Adhesin in an in vitro model of rabbit respiratory epithelium. Vet. Med. Int..

[cit14] Boyce J. D., Adler B. (2000). The capsule is a virulence determinant in the pathogenesis of Pasteurella multocida M1404 (B:2). Infect. Immun..

[cit15] Harper M., Cox A. D., St Michael F., Wilkie I. W., Boyce J. D., Adler B. (2004). A heptosyltransferase mutant of Pasteurella multocida produces a truncated lipopolysaccharide structure and is attenuated in virulence. Infect. Immun..

[cit16] Harper M., Cox A., St Michael F., Parnas H., Wilkie I., Blackall P. J. (2007). *et al.*, Decoration of Pasteurella multocida lipopolysaccharide with phosphocholine is important for virulence. J. Bacteriol..

[cit17] Horadagoda N. U., Hodgson J. C., Moon G. M., Wijewardana T. G., Eckersall P. D. (2002). Development of a clinical syndrome resembling haemorrhagic septicaemia in the buffalo following intravenous inoculation of Pasteurella multocida serotype B:2 endotoxin and the role of tumour necrosis factor-α. Res. Vet. Sci..

[cit18] Wang Z., Li J., Altman E. (2006). Structural characterization of the lipid A region of Aeromonas salmonicida subsp. salmonicida lipopolysaccharide. Carbohydr. Res..

[cit19] Erridge C., Bennett-Guerrero E., Poxton I. R. (2002). Structure and function of lipopolysaccharides. Microbes Infect..

[cit20] Da Silva Correia J., Soldau K., Christen U., Tobias P. S., Ulevitch R. J. (2001). Lipopolysaccharide is in close proximity to each of the proteins in its membrane receptor complex. J. Biol. Chem..

[cit21] Murphy K. and Weaver C., The induced responses of innate immunity, Garland Science/Taylor and Francis Group, LLC, New York, NY, USA, 9th edn, Janeway's Immunobiology, 2017, p. 924

[cit22] Darveau R. P., Pham T.-T. T., Lemley K., Reife R. A., Bainbridge B. W., Coats S. R. (2004). *et al.*, Porphyromonas gingivalis lipopolysaccharide contains multiple lipid A species that functionally interact with both toll-like receptors 2 and 4. Infect. Immun..

[cit23] Molinaro A., Holst O., Lorenzo F. D., Callaghan M., Nurisso A., D'Errico G. (2015). *et al.*, Chemistry of lipid A: At the heart of innate immunity. Chem.–Eur. J..

[cit24] Crittenden C. M., Akin L. D., Morrison L. J., Trent M. S., Brodbelt J. S. (2016). Characterization of lipid A variants by energy-resolved mass spectrometry: Impact of acyl chains. J. Am. Soc. Mass Spectrom..

[cit25] Kabanov D. S., Prokhorenko I. R. (2010). Structural analysis of lipopolysaccharides from gram-negative bacteria. Biochemistry.

[cit26] Jeyaretnam B., Glushka J., Kolli V. S. K., Carlson R. W. (2002). Characterization of a Novel Lipid-A from Rhizobium Species Sin-1 a Unique Lipid-A Structure That is Devoid of Phosphate and has a Glycosyl Backbone Consisting of Glucosamine and 2-Aminogluconic Acid. J. Biol. Chem..

[cit27] Que N. L. S., Lin S., Cotter R. J., Raetz C. R. H. (2008). Purification and mass spectrometry of six Lipid A Species from the bacterial endosymbiont Rhizobium etli. J. Biol. Chem..

[cit28] Zhou Z., Lin S., Cotter R. J., Raetz C. R. H. (1999). Lipid A modifications characteristic of Salmonella typhimurium are induced by NH_4_ VO_3_ in Escherichia coli K12. J. Biol. Chem..

[cit29] Wang X., Quinn P. J. (2010). Lipopolysaccharide: diosynthetic pathway and structure modification. Prog. Lipid Res..

[cit30] Caroff M., Karibian D., Cavaillon J. M., Haeffner-Cavaillon N. (2002). Structural and functional analyses of bacterial lipopolysaccharides. Microbes Infect..

[cit31] Lodowska J., Wolny D., Weglarz L., Dzierzewicz Z. (2007). The structural diversity of lipid A from Gram-negative bacteria. Postepy Hig. Med. Dosw..

[cit32] Alexander C., Zähringer U. (2002). Chemical structure of lipid A - the primary immunomodulatory center of bacterial lipopolysaccharides. Trends Glycosci. Glycotechnol..

[cit33] Geurtsen J., Steeghs L., Hove J. t., Ley P. V. D., Tommassen J. (2005). Dissemination of Lipid A Deacylases (PagL) among Gram-negative bacteria. J. Biol. Chem..

[cit34] Mills G., Dumigan A., Kidd T., Hobley L., Bengoecheaa J. A. (2017). Identification and characterization of two Klebsiella pneumoniae lpxL lipid A late Acyltransferases and their role in virulence. Infect. Immun..

[cit35] Lukasiewicz J., Jachymek W., Niedziela T., Kenne L., Lugowski C. (2010). Structural analysis of the lipid A isolated from Hafnia alvei 32 and PCM 1192 lipopolysaccharides. J. Lipid Res..

[cit36] Jo S.-H., Park H.-G., Song W.-S., Kim S.-M., Kim E.-J., Yang Y.-H. (2019). *et al.*, Structural characterization of phosphoethanolamine-modified lipid A from probiotic: Escherichia coli strain Nissle 1917. RSC Adv..

[cit37] Needham B. D., Trent M. S. (2013). Fortifying the barrier: the impact of lipid A remodelling on bacterial pathogenesis. Nat. Rev. Microbiol..

[cit38] Raetz C. R. H., Reynolds C. M., Trent M. S., Bishop R. E. (2007). Lipid A modificaton system in Gram-negative bacteria. Annu. Rev. Biochem..

[cit39] St Michael F., Vinogradov E., Li J., Cox A. D. (2005). Structural analysis of the lipopolysaccharide from Pasteurella multocida genome strain Pm70 and identification of the putative lipopolysaccharide glycosyltransferases. Glycobiology.

[cit40] Steimle A., Autenrieth I. B., Frick J. S. (2016). Structure and function: Lipid A modifications in commensals and pathogens. Int. J. Med. Microbiol..

[cit41] Reife R. A., Coats S. R., Al-Qutub M., Dixon D. M., Braham P. A., Billharz R. J. (2006). *et al.*, Porphyromonas gingivalis lipopolysaccharide lipid A heterogeneity: differential activities of tetra- and penta-acylated lipid A structures on E-selectin expression and TLR4 recognition. Cell. Microbiol..

[cit42] Domon B., Costello C. E. (1988). A systematic nomenclature for carbohydrate fragmentations in FAB-MS/MS spectra of glycoconjugates. Glycoconjugate J..

[cit43] Morrison L. J., Parker W. R., Holden D. D., Henderson J. C., Boll J. M., Trent M. S. (2016). *et al.*, UVliPiD: A UVPD-based hierarchical approach for de novo characterization of lipid A structures. Anal. Chem..

[cit44] Kussak A., Weintraub A. (2002). Quadrupole ion-trap mass spectrometry to locate fatty acids on lipid A from Gram-negative bacteria. Anal. Biochem..

[cit45] CABI , Detailed coverage of invasive species threatening livelihoods and the environment worldwide, data sheet on Pasteurella multocida infections, CABI Invasive Species Compendium, 2020, available from https://www.cabi.org/isc/datasheet/70916#todistribution, last updated on 10th Jan, 2020

[cit46] Bader M. W., Sanowar S., Daley M. E., Schneider A. R., Cho U., Xu W. (2005). *et al.*, Recognition of antimicrobial peptides by a bacterial sensor kinase. Cell.

[cit47] Trent M. S., Ribeiro A. A., Lin S., Cotter R. J., Raetz C. R. H. (2001). An inner membrane enzyme in Salmonella and Escherichia coli that transfers 4-amino-4-deoxy-L-arabinose to lipid A: induction on polymyxin-resistant mutants and role of a novel lipid-linked donor. J. Biol. Chem..

[cit48] Zhou Z., Ribeiro A. A., Lin S., Cotter R. J., Miller S. I., Raetz C. R. H. (2001). Lipid A modifications in polymyxin-resistant Salmonella typhimurium: PmrA-dependent 4-amino-4-deoxy-L-arabinose, and phosphoethanolamine incorporation. J. Biol. Chem..

[cit49] McCoy A. J., Liu H., Falla T. J., Gunn J. S. (2001). Identification of Proteus mirabilis mutants with increased sensitivity to antimicrobial peptides. Antimicrob. Agents Chemother..

[cit50] Beceiro A., Llobet E., Aranda J., Bengoechea J. A., Doumith M., Hornsey M. (2011). *et al.*, Phosphoethanolamine modification of lipid A in colistin-resistant variants of Acinetobacter baumannii mediated by the pmrAB two-component regulatory system. Antimicrob. Agents Chemother..

[cit51] Ernst R. K., Yi E. C., Guo L., Lim K. B., Burns J. L., Hackett M., Miller S. I. (1999). Specific lipopolysaccharide found in cystic fibrosis airway Pseudomonas aeruginosa. Science.

[cit52] Bishop R. E. (2005). The lipid A palmitoyltransferase PagP: molecular mechanisms and role in bacterial pathogenesis. Mol. Microbiol..

[cit53] Loppnow H., Brade L., Brade H., Rietschel E. T., Kusumoto S., Shiba T. (1986). *et al.*, Induction of human interleukin 1 by bacterial and synthetic lipid A. Eur. J. Immunol..

[cit54] Qureshis N., Takayamas K., Hellerq D., Fenselauq C. (1983). Position of ester groups in the lipid A backbone of lipopolysaccharides obtained from Salmonella typhimurium. J. Biol. Chem..

[cit55] Hankins J. V., Madsen J. A., Giles D. K., Brodbelt J. S., Trent M. S. (2012). Amino acid addition to Vibrio cholerae LPS establishes a link between surface remodeling in Gram-positive and Gram-negative bacteria. Proc. Natl. Acad. Sci. U. S. A..

[cit56] Coats S. R., Berezow A. B., To T. T., Jain S., Bainbridge B. W., Banani K. P. (2011). *et al.*, The lipid A phosphate position determines differential host toll-like receptor 4 responses to phylogenetically related symbiotic and pathogenic bacteria. Infect. Immun..

[cit57] Gaddy J. A., Radin J. N., Cullen T. W., Chazin W. J., Skaar E. P., Trent M. S. (2015). *et al.*, Helicobacter pylori resists the antimicrobial activity of calprotectin via lipid A modification and associated biofilm formation. mBio.

[cit58] Phillips N. J., Adin D. M., Stabb E. V., McFall-Ngai M. J., Apicella M. A., Gibson B. W. (2011). The lipid A from Vibrio fischeri lipopolysaccharide: a unique structure bearing a phosphoglycerol moiety. J. Biol. Chem..

[cit59] Feng G., Hao Y., Wu L., Chen S. (2020). A visible-light activated [2 + 2] cycloaddition reaction enables pinpointing carbon-carbon double bonds in lipids. Chem. Sci..

[cit60] Townsend K. M., Frost A. J., Lee C. W., Papadimitriou J. M., Dawkins H. J. S. (1998). Development of PCR assays for species- and type-specific identification of Pasteurella multocida isolates. J. Clin. Microbiol..

[cit61] Masoud H., Martin A., Thibault P., Richard Moxon E., Richards J. C. (2003). Structure of extended lipopolysaccharide glycoforms containing two globotriose units in Haemophilus influenzae serotype b strain RM7004. Biochemistry.

[cit62] Westphal O. and Jann K., Bacterial lipopolysaccharides: extraction with phenol–water and further applications of procedure, ed. R. L. Whisler, Methods Carbohydr Chem, Acad Press Inc, New York, 1965, vol. 5, pp. 83–91

[cit63] Guard-Petter J., Lakshmi B., Carlson R., Ingram K. (1995). Characterization of lipopolysaccharide heterogeneity in Salmonella enteritidis by an improved gel electrophoresis method. Appl. Environ. Microbiol..

[cit64] Mahat R., Seebart C., Basile F., Ward N. L. (2016). Global and targeted lipid analysis of Gemmata obscuriglobus reveals the presence of lipopolysaccharide, a signature of the classical Gram-negative outer membrane. J. Bacteriol..

[cit65] Chu C., Liu B., Watson D., Szu S., Bryla D., Shiloach J. (1991). *et al.*, Preparation, characterization, and immunogenicity of conjugates composed of the O-specific polysaccharide of Shigella dysenteriae type 1 (Shiga's bacillus) bound to tetanus toxoid. Infect. Immun..

[cit66] Michael F. St, Li J., Cox A. D. (2005). Structural analysis of the core oligosaccharide from Pasteurella multocida strain X73. Carbohydr. Res..

[cit67] Michael F. St, Li J., Vinogradov E., Larocque S., Harper M., Cox A. D. (2005). Structural analysis of the lipopolysaccharide of Pasteurella multocida strain VP161: identification of both Kdo-P and Kdo-Kdo species in the lipopolysaccharide. Carbohydr. Res..

[cit68] Harper M., Michael F. St, Vinogradov E., John M., Boyce J. D., Adler B., Cox A. D. (2012). Characterization of the lipopolysaccharide from Pasteurella multocida Heddleston serovar 9: Identification of a proposed bi-functional dTDP-3-acetamido-3,6-dideoxy-α-D-glucose biosynthesis enzyme. Glycobiology.

[cit69] Sarangi L. N., Thomas P., Gupta S. K., Priyadarshini A., Kumar S., Nagaleekar V. K., Kumar A., Singh V. P. (2015). Virulence gene profiling and antibiotic resistance pattern of Indian isolates of Pasteurella multocida of small ruminant origin. Comp. Immunol. Microbiol. Infect. Dis..

